# Enzymatic Synthesis of Resveratrol α-Glucoside by Amylosucrase of *Deinococcus geothermalis*

**DOI:** 10.4014/jmb.2108.08034

**Published:** 2021-09-26

**Authors:** Keumok Moon, Seola Lee, Hyunsu Park, Jaeho Cha

**Affiliations:** 1 Microbiological Resource Research Institute, Pusan National University, Busan 46241, Republic of Korea; 2 Department of Microbiology, Pusan National University, Busan 46241, Republic of Korea

**Keywords:** Amylosucrase, bioavailability, antioxidant activity, melanogenesis, resveratrol, enzymatic glycosylation

## Abstract

Glycosylation of resveratrol was carried out by using the amylosucrase of *Deinococcus geothermalis*, and the glycosylated products were tested for their solubility, chemical stability, and biological activities. We synthesized and identified these two major glycosylated products as resveratrol-4′-*O*-α-glucoside and resveratrol-3-*O*-α-glucoside by nuclear magnetic resonance analysis with a ratio of 5:1. The water solubilities of the two resveratrol-α-glucoside isomers (α-piceid isomers) were approximately 3.6 and 13.5 times higher than that of β-piceid and resveratrol, respectively, and they were also highly stable in buffered solutions. The antioxidant activity of the α-piceid isomers, examined by radical scavenging capability, showed it to be initially lower than that of resveratrol, but as time passed, the α-piceid isomers’ activity reached a level similar to that of resveratrol. The α-piceid isomers also showed better inhibitory activity against tyrosinase and melanin synthesis in B16F10 melanoma cells than β-piceid. The cellular uptake of the α-piceid isomers, which was assessed by ultra-performance liquid chromatography (UPLC) analysis of the cell-free extracts of B16F10 melanoma cells, demonstrated that the glycosylated form of resveratrol was gradually converted to resveratrol inside the cells. These results indicate that the enzymatic glycosylation of resveratrol could be a useful method for enhancing the bioavailability of resveratrol.

## Introduction

During the past several decades, numerous studies have shown the negative correlation between wine consumption and the risk of cardiovascular diseases such as atherosclerosis, that is, the “French paradox” [1, 2]. Resveratrol (*trans*-3,5,4′-trihydroxystilbene) is a polyphenolic compound present in grapes and blueberries and has strong anti-oxidative activities [3, 4]. This compound also exhibits an inhibitory effect on melanin synthesis, anti-inflammatory activity, anti-cancer, and osteoblast differentiation [4-6]. Numerous data are available on the beneficial effects of resveratrol [3, 7-9]. Therefore, resveratrol is commercially used as a dietary supplement in the pharmaceutical and food industries due to its biological potential.

Although resveratrol has beneficial health effects, its low bioavailability and chemical instability may limit its practical application in these industries. The oral absorption of resveratrol (25 mg oral dose) in humans is approximately 75% [10, 11]. This value is higher than that of any other dietary polyphenols despite its water insolubility. However, the extensive metabolism in the intestine and liver results in an oral bioavailability considerably less than 1% [10, 12, 13]. Various methods have been used to improve the aqueous solubility and bioavailability of resveratrol, and as a result, numerous resveratrol derivatives have been developed [14, 15]. Glycosylation is a popular method used to enhance water solubility by adding a sugar moiety to compounds by means of an enzymatic reaction [16-19]. Although the chemical synthesis of glycosides is reasonable, it generates considerable amounts of waste products and environmental pollution. The higher selectivity and specificity of enzymatic synthesis will enable the synthesis of glycosylated products more easily compared with chemical synthesis.

Phenolic compounds, such as catechin, naringin, baicalein, and salicin, are commonly glycosylated by bacterial glucosidases and/or glycosyltransferase [16, 19-21]. Enzymatic synthesis is highly applicable for the production of natural compound analogues with improved solubility [17, 22]. Glycosylation is also used to ameliorate unstable substances caused by oxidation. The half-life (*t_1/2_*) of resveratrol in the reaction of polyphenol oxidases is three times shorter than that of piceid, which is a natural resveratrol glucoside. The attachment of a glucose residue poses a steric hindrance that inhibits the complete enzymatic oxidation of piceid [23]. Enzymatically synthesized resveratrol glucosides are expected to maintain their beneficial antioxidant activities and provide increased bioavailability in humans compared with resveratrol.

In this study, resveratrol glucosides were synthesized through transglycosylation by the amylosucrase from *Deinococcus geothermalis* to improve the bioavailability of resveratrol. The glycosylation was successfully conducted with resveratrol as the acceptor and sucrose as the donor. The molecular structures of the glycosylated compounds, that is, the resveratrol glucosides, were identified, and their water solubility, oxidative stability, and biological activities were examined compared with resveratrol and β-piceid, a natural resveratrol glucoside.

## Materials and Methods

### Chemicals and Reagents

Sucrose, resveratrol, and β-piceid were purchased from Sigma-Aldrich (USA). Water, methanol, and acetonitrile (high-performance liquid chromatography grade) were purchased from Burdick & Jackson (USA) for purification of transfer products. Dulbecco’s modified Eagle medium (DMEM) was purchased from WelGENE Inc. (Korea). Fetal bovine serum (FBS) and antibiotics (penicillin/streptomycin) were purchased from Gibco (Thermo Fisher Scientific, USA). Diphenyl-2-picrylhydrazyl (DPPH) and 2,4,6-tris(2-pyridyl)-S-triazine (TPTZ) were purchased from Sigma-Aldrich. All other chemicals were of reagent grade and purchased from Sigma-Aldrich. The recombinant amylosucrase from *D. geothermalis* (DGAS) was prepared in *E. coli* as previously described [16].

### Enzymatic Synthesis of Resveratrol Glucosides with DGAS

The substrate solution in 50 mM Tris-HCl buffer (pH 8.0) containing 5 mM resveratrol, 10 mM sucrose, and 20% dimethylsulfoxide (DMSO) was preincubated at 35°C for 10 min, followed by the addition of DGAS (0.1 mg/ml). The incubation of the reaction mixture was continued for 12 h [16]. The reaction was stopped by boiling for 10 min and placing the mixture tube in ice. The amount of synthesized resveratrol glucosides was determined by thin-layer chromatography (TLC) and ultra-performance liquid chromatography (UPLC) analyses.

### TLC and UPLC Analyses

TLC was performed using silica gel 60 and silica gel RP-18 F254S (Merck KGaA, Germany) to identify the respective resveratrol glucosides, and the plates were developed in a developing solution containing *n*-butanol/ethanol/water (5:3:2, v/v/v) and methanol/water (5:5, v/v) for silica gel 60 and silica gel RP-18 F254S, respectively. The developed TLC plates were dried completely at room temperature and then visualized by dipping in a solution containing 1% (w/v) *N*-(1-naphtyl)-ethylenediamine and 20% (v/v) sulfuric acid in methanol followed by heating at 110°C for 10 min.

UPLC analysis was performed on an Acquity H-Class UPLC system (Waters, Ireland), which comprises a photo diode array eλ detector. The separation of resveratrol and resveratrol glucosides was carried out by reversed-phase LC using a BEH C_18_ (2.1 mm × 50 mm id, particle size: 1.7 μm) column (Waters). The column temperature was 40°C, the flow rate was 0.6 ml/min, and the injection volume was 2 μl. The resveratrol and its glucosides were observed at 306 nm, at which resveratrol showed an absorbance maxima. Solvent A was 0.1% (v/v) formic acid in water, and solvent B was 0.02% (v/v) formic acid in 80% (v/v) acetonitrile. The gradient conditions were as follows: 82% A over 0.68 min, 82% to 77% A over 0.48 min, 77% to 75.5% A over 0.27 min, 75.5% to 68.5% A over 0.41 min, 68.5% to 0% A over 0.2 min, 0% A over 0.34 min, and then 82% A for 2.62 min.

### Purification of Resveratrol Glucosides

The resveratrol glucosides were purified in accordance with the method described by Moon *et al*. [17]. In brief, the resveratrol glucosides were separated by Strata C_18_-T cartridge (Phenomenex, USA) and preparative LC (Prep LC) equipped with an ultraviolet (UV) detector (JAI, Japan). A C_18_-T cartridge, which was previously activated by methanol and water, was used to absorb the resveratrol glucosides in the reaction mixture and to eliminate sugar and salts. The reaction mixture was filtered by a 0.45 μm syringe filter (Germany) and subjected to a C_18_-T cartridge. After washing with water, the elution of resveratrol glucosides was carried out with methanol. The main resveratrol glucosides in methanol were purified using a W252 polymeric gel filtration column (2 cm × 50 cm, JAI) in the Prep LC. The mobile phase was 100% methanol at a flow rate of 3 ml/min. The fractions corresponding to the detected peaks were collected and lyophilized. The purity of each sample was confirmed using UPLC analysis.

### Nuclear Magnetic Resonance (NMR) Analysis

The ^1^H and ^13^C NMR spectra of purified resveratrol glucosides were obtained with a Varian Inova AS 400 MHz NMR spectrometer (Varian, USA). The sample was dissolved in DMSO-*d*_6_ at 24°C with tetramethylsilane as the chemical shift reference.

### Water Solubility Determination

To maximize the solubility of each compound, we suspended the excess amounts of resveratrol, β-piceid, and resveratrol glucosides in 200 μl distilled water in a microcentrifuge tube and then sonicated the tube for 1 h at room temperature using an ultrasonic cleaner (JAC-4020, Kodo, Korea) [17]. Each sample was centrifuged at 16,000 g for 30 min. Subsequently, the supernatant of each sample was filtered, and the concentration of the compound in the supernatant as a water-soluble component was estimated by UPLC analysis. Resveratrol and β-piceid were used as standards, and the water solubility was expressed as g/l.

### Stability against Oxidative Degradation

Resveratrol, β-piceid, and resveratrol glucosides were dissolved in buffer solutions of various pH values (hydrochloric acid, pH 2.0; phosphate-buffered saline (PBS), simulated body fluid (SBF), pH 7.4; Tris-HCl, pH 9.0, and DMEM to reach a final concentration of 10 mM. The solutions were then incubated at 37°C, and an aliquot (300 μl) of the reaction mixture was extracted at different time points up to 72 h, filtered, and analyzed by UPLC.

### Determination of Antioxidant Activities

DPPH free-radical scavenging capability and ferric reducing antioxidant power (FRAP) assay results were evaluated as described previously by Moon *et al*. [24]. Hydroxy radical-scavenging assay was measured as described by Su *et al*. [3]. Vitamin C was used as a positive control. Each assay was measured from 1 min to 30 h. The DPPH radical-scavenging activity was calculated as follows: DPPH radical-scavenging activity (%) = (1-A_sample_/A_blank_) × 100, where A_sample_ is the absorbance in the presence of the sample, and A_blank_ is the absorbance of the blank. Hydroxy radical-scavenging activity was calculated in accordance with the following formula: Hydroxy radical scavenging activity (%) = (A_sample_-A_control_)/(A_blank_-A_control_) × 100, where A_sample_ is the absorbance in the presence of the sample, A_control_ is the absorbance of the blank with H_2_O_2_, and A_blank_ is the absorbance of the blank without H_2_O_2_.

### Determination of Anti-Melanogenesis Activity

**Cell culture and cell viability assay.** B16F10 melanoma cells were maintained in DMEM supplemented with 10% FBS, 100 U/ml penicillin, and 100 μg/ml streptomycin. The cells were cultured at 37°C under 5% CO_2_ in a humidified culture chamber. Cell viability was determined by 3-(4,5-dimethythiazol-2-yl)-2,5-diphenyl tetrazolium bromide (MTT) assay. The cells (6 × 10^3^ cells/well) were seeded into 96-well plates and cultured for 24 h. The cells were then treated with resveratrol, β-piceid, or resveratrol glucosides at various concentrations. After 24 h, the cells were washed with PBS, MTT reagent (0.5 mg/ml) was added to each well, and the cells were incubated at 37°C for 1 h. The supernatant in each well was discarded for the treatment with DMSO, which was used to dissolve the formazan crystals formed from viable cells. After the DMSO treatment, the absorbance of each well was measured at 595 nm using a microplate reader (Epoch, Biotek, USA).

### Determination of Cellular Tyrosinase Activity and Melanin Content

Cellular tyrosinase activity was measured following the method described by Park *et al*. with slight modifications [25]. The cells (4 × 10^4^ cells/well) were seeded into 24-well plates and cultured for 24 h. The cells were exposed to resveratrol, β-piceid, or resveratrol glucosides in the presence or absence of 100 nM α-melanocyte stimulating hormone (α-MSH) for 48 h. The cells were then washed with PBS and lysed with a lysis buffer. The lysates were then clarified by centrifugation at 16,000 g for 15 min at 4°C. The protein concentration was determined by Bradford method (Bio-Rad Laboratories Inc., USA) using bovine serum albumin as a standard. The reaction mixture consisting of 50 μg protein (adjusted to 100 μl with 0.1 M sodium phosphate, pH 6.8) and 100 μl 10 mM L- 3,4-dihydroxyphenylalanine was added to each well of the 96-well plates. After incubation at 37°C for 1 h, the absorbance was measured at 490 nm using a microplate reader.

Cellular melanin content was determined by the method of Park *et al*. with minor modifications [25]. The cells (5 × 10^4^ cells/well) were seeded into 6-well plates and cultured for 24 h. The cells were treated with resveratrol, β-piceid, or resveratrol glucosides for 72 h. Then, the cells were washed with PBS and lysed with lysis buffer, and the supernatant was removed by centrifugation at 16,000 g for 15 min at 4°C. The protein concentration of the supernatant was determined by the Bradford method. The pellet was dissolved in 1 M NaOH and measured at 405 nm using a microplate reader.

### Cellular Uptake Assay

The cellular uptake assay was conducted following the method described by Moon *et al*. with minor modification [17]. The B16F10 cells (2 × 10^5^ cells/well) were seeded into 6-well plates. The cells were cultured for 24 h, and then, resveratrol (20 μM), β-piceid, or resveratrol glucosides (100 μM) was added. After 24 h of incubation, the medium was removed, and the cells were washed with PBS and then scraped off. After centrifugation, the cell pellet was resuspended with 100 μl of 10 mM HCl and lysed by freezing and thawing using liquid nitrogen three times. The lysate was centrifuged at 16,000 g for 10 min and filtered by a 0.2 μm syringe filter. The cell extracts were analyzed by UPLC.

### Statistical Analysis

All experiments were performed in triplicate. Data were analyzed using SigmaPlot software (version 12.5, Systat, USA) and expressed as mean ± SD. The data were analyzed by one-way analysis of variance, and the mean value was considered to be significantly different at *p* < 0.05, *p* < 0.005, and *p* < 0.001.

## Results

### Synthesis and Identification of Resveratrol Glucosides by DGAS

Resveratrol was glycosylated by DGAS using resveratrol as the acceptor and sucrose as the donor molecule, and the reaction products were detected by TLC and UPLC analyses. On the TLC plate, the spots corresponding to sucrose and fructose were visible, whereas the other spots were possibly the produced glucoside derivatives (Fig. S1). The sugars included in the reaction products were first removed by a reversed-phase C_18_-T cartridge. Then, the mixture of resveratrol glucosides was separated by using a W252 polymeric gel-filtration column through Prep LC. The separated resveratrol glucosides were analyzed by TLC and UPLC.

**Fig. 1:**
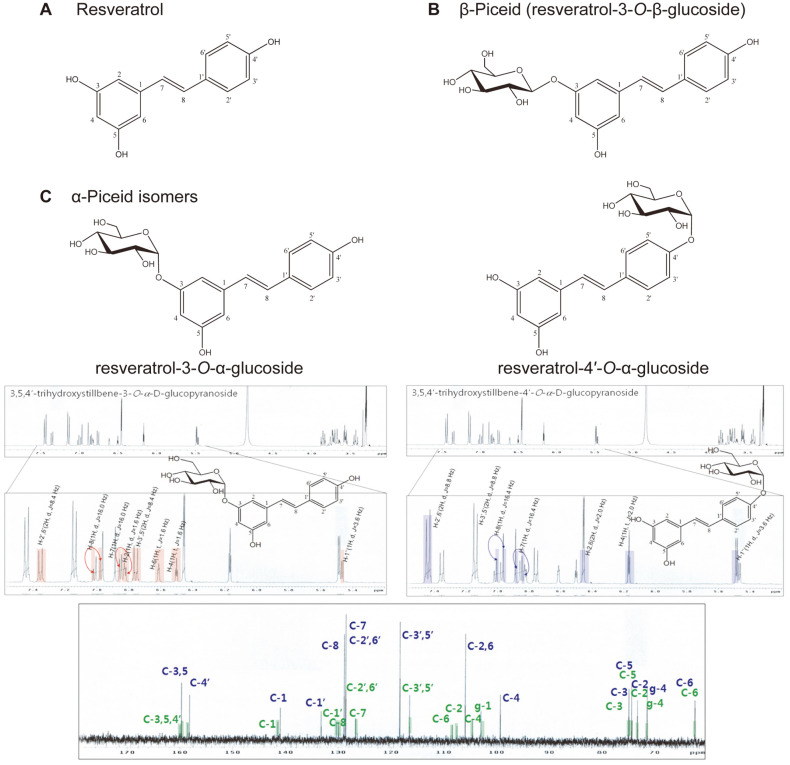
Structure of resveratrol and the glycosylated products. (**A**) resveratrol, (**B**) β-piceid, and (**C**) ^1^H and ^13^C NMR spectra of the major resveratrol transfer products, α-piceid isomers.

The three resveratrol glucosides were detected by TLC analysis (Fig. S1, lanes 6–8). The major compound (lane 8) among three resveratrol glucosides was isolated, and the silica gel RP-18 F254S revealed it to be two compounds. The molecular structure of these two compounds was confirmed by NMR analysis. The ^1^H and ^13^C NMR spectra of these compounds were compared with those of resveratrol. Two anomer proton signals were detected, and the bonds between the glucose moiety and aglycon resveratrol were determined to be α-glycosidic linkages based on the coupling constant (^1^H, d, *J* = 3.6 Hz) in the ^1^H NMR spectrum. The carbon signal of the NMR results indicated that DGAS transferred a glucose residue of sucrose to the C-4′ or C-3 position in the resveratrol skeleton. Based on the ^1^H and ^13^C spectra of the major resveratrol glucosides, two isomers were detected and determined to be resveratrol-4′-*O*-α-glucoside (R1) and resveratrol-3-*O*-α-glucoside (R2) (Fig. 1). The presence of two resveratrol-α-glucoside isomers (α-piceid isomers) was confirmed by TLC silica gel 60 F254S plate, which is known as an octadecyl silica TLC plate showing two TLC spots (Fig. S1, lane 9). Likewise, the other resveratrol glucosides synthesized by DGAS were determined to be resveratrol-4′-*O*-α-maltoside and resveratrol-3-*O*-α-maltoside with α-1,4-glucosidic linkage (data not shown).

The major resveratrol-α-glucoside isomers (R1 and R2) were analyzed by UPLC analysis. Under the reversed-phase UPLC condition, two isomer peaks were detected by a UV detector (Fig. S2), and R1 was obtained as the major resveratrol glucoside. The ratio of the peak areas for R1 and R2 in the UPLC profile was approximately 5:1. The conversion yields of the two compounds from resveratrol were 38.7% (R1) and 6.8% (R2).

### Physicochemical Properties of α-Piceid Isomers

Phenolic compounds have been studied to increase their applicability in pharmaceutical or cosmetic preparations by improving their low solubility or chemical stability [16-19, 21]. The relative polarity of the α-piceid isomers was evaluated by comparing their retention times with those of β-piceid and resveratrol by UPLC analysis combined with a C_18_ column. The retention times for R1, R2, β-piceid, and resveratrol were 0.950, 1.254, 1.255, and 1.904 min, respectively (Fig. S2). Thus, R1 is a more polar compound than R2, β-piceid, and resveratrol. The retention times of R2 and β-piceid were almost the same, and no difference was observed due to the α- or β-binding of the glucosyl moiety at the C-3 position of resveratrol. This finding implies that the attachment of a glucosyl residue to the C-4′ position of resveratrol resulted in better polarity than attaching it to the C-3 position of resveratrol. The water solubilities of resveratrol, β-piceid, R1, and R2 were 0.036 (157.7 μM), 0.137 (350.92 μM), 0.515 (1.319 mM), and 0.471 g/L (1.206 mM), respectively. The water solubility of the α-piceid isomers was about 3.6 and 13.5 times higher than that of β-piceid and resveratrol, respectively.

In general, stilbenes, which make up the skeleton of resveratrol, undergo rapid autoxidative degradation. Thus, their half-lives (*t_1/2_*) in buffered aqueous solutions are as short as 10 h [26, 27]. The oxidative stability of resveratrol and piceid isomers was determined by their half-lives in various solutions. Resveratrol decomposed rapidly at a high pH, whereas it was very stable in acidic conditions, with a half-life of >72 h. By contrast, both α-piceid isomers (R1 and R2) and β-piceid were stable in all the tested solutions (Table 1). The improved chemical stability of the piceid isomers was due to the glycosylation of the oxidative group of resveratrol, which prevented its oxidative degradation after incubation for 72 h at alkaline pH [17, 19].

### Effects of α-Piceid Isomers on Antioxidant Activities

**Fig. 2:**
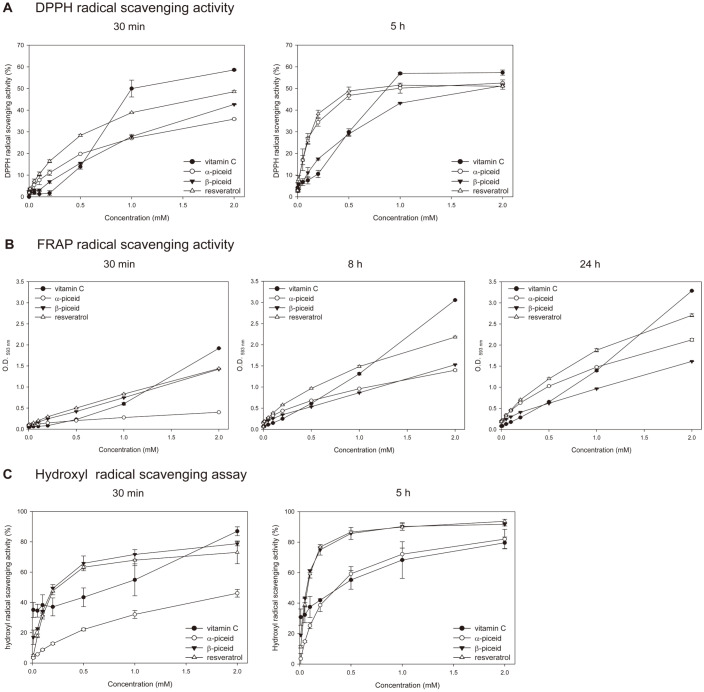
Determination of antioxidant activity of vitamin C, resveratrol, β-piceid and α-piceid isomers by (**A**) DPPH, (**B**) FRAP, and (**C**) hydroxyl radical scavenging method. Each compound was used at concentration ranges between 0 to 2 mM. Values are expressed as mean ± SD from triplicates. **A**: DPPH radical scavenging activity at 30 min and 5 h, **B**: FRAP radical scavenging activity at 30 min, 8 h, and 24 h, **C**: hydroxyl radical scavenging activity at 30 min and 5 h.

The antioxidant activities of resveratrol, β-piceid, and the α-piceid isomers were measured by DPPH, FRAP, and hydroxyl radical scavenging assay (Figs. 2, S3). These antioxidant assays have been widely used to determine the antioxidant capacities of natural products and are highly reproducible test methods [28]. In the DPPH radical-scavenging assay, vitamin C rapidly scavenged the DPPH radical within 30 min, whereas stilbenes exhibited maximal scavenging activities within 2–5 h (Figs. 2A, S3A). Notably, the DPPH radical-scavenging activity of the α-piceid isomers was similar to that of resveratrol. The EC_50_ values of resveratrol, β-piceid, and α-piceid isomers were 0.5232, 0.4412, and 0.9242 mM at 30 min, respectively. Meanwhile, the values were 0.1181 (resveratrol), 0.6992 (β-piceid), and 0.1300 (α-piceid isomers) mM at 5 h. The radical-scavenging activity of the α-piceid isomers was initially lower than that of β-piceid, but as time passed, the radical-scavenging activity of the α-piceid isomers became superior to that of β-piceid.

Vitamin C, resveratrol, and β-piceid removed the FRAP radical quickly (Figs. 2B, S3B). Similar to the DPPH radical-scavenging activity, the FRAP radical-scavenging activity of α-piceid isomers was initially lower than that of β-piceid but increased over time. The radical-scavenging activity of the α-piceid isomers was lower than that of β-piceid at 30 min, attained a value similar at 8 h, and was higher at 24 h. Interestingly, the reaction of FRAP radical scavenging by β-piceid showed no increase over time, unlike other samples.

Contrary to the results of the DPPH radical-scavenging assay, the hydroxyl radical-scavenging activity of β-piceid was similar to that of resveratrol, and the activity of the α-piceid isomers was lower than that of β-piceid (Figs. 2C, S3C). The EC_50_ values of resveratrol, β-piceid, and α-piceid isomers were 0.1382, 0.2151, and 1.5326 mM at 30 min, respectively. The values were 0.0671 (resveratrol), 0.0537 (β-piceid), and 0.2755 (α-piceid isomers) mM at 5 h.

### Effects of α-Piceid Isomers on Melanogenesis

Melanoma cells have been used to evaluate the inhibition of melanin synthesis and cell toxicity [29]. To investigate the structure–activity relationship of resveratrol glucosides for the antiproliferative activity on B16F10 melanoma cells, we examined resveratrol and the piceid isomers by MTT assay. B16F10 cells were incubated with the indicated concentration of the compounds for 1 day. Resveratrol inhibited the growth of B16F10 cells in a dose-dependent manner, but neither β-piceid nor the α-piceid isomers affected the proliferation of B16F10 cells up to 100 μM. However, the α-piceid isomers inhibited the growth of B16F10 cells at concentrations above 100 μM (Fig. S4).

**Fig. 3:**
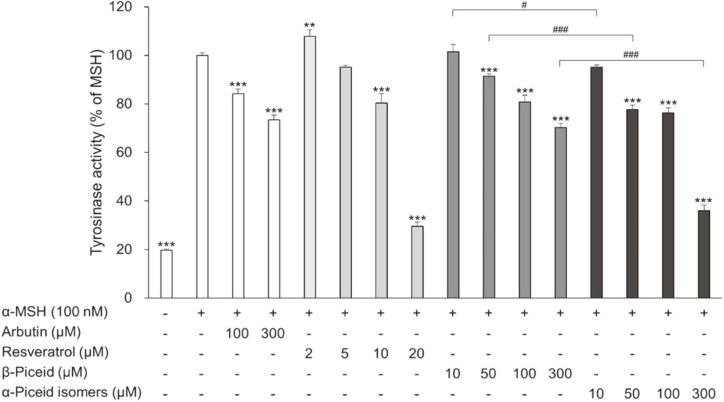
Effect of resveratrol, β-piceid and α-piceid isomers on cellular tyrosinase activity in α-MSHstimulated B16F10 melanoma cells. The cells were treated with α-MSH alone or together with arbutin, resveratrol, β- piceid, or α-piceid isomers for 48 h and then cellular tyrosinase activity was measured. Significant differences were compared with α-MSH treated cells: **p < 0.005 and ***p < 0.001, significant differences were also observed between β-piceid and α-piceid isomers: #*p* < 0.05 and ###*p* < 0.001.

Melanin is synthesized through several steps in the cells and is influenced by various factors. Stilbenes downregulate melanogenesis-related proteins, such as tyrosinase, tyrosinase-related protein (TRYP) 1, TRYP2, and microphthalmia-associated transcription factor, in melanoma cells [30-32]. Resveratrol also exhibited an inhibitory effect against mushroom tyrosinase (EC1.14.18.1) through a *k*_cat_ inhibition [33]. Piceid, which has been used in cosmetics, inhibits tyrosinase activity [34]. To confirm any dissimilarity in the whitening effects due to their structural difference, we examined the effects of the α-piceid isomers on cellular tyrosinase activity and melanin synthesis in B16F10. Arbutin, a representative whitening agent, was evaluated as a positive control. Resveratrol inhibited the tyrosinase activity at concentrations above 10 μM. Compared with cells treated with MSH alone, resveratrol suppressed the tyrosinase activity by 19.6% and 70.5% in cells treated at concentrations of 10 and 20 μM, respectively (Fig. 3). Unlike resveratrol, the piceid isomers inhibited the tyrosinase activity at concentrations above 50 μM. Arbutin, β-piceid, and the α-piceid isomers at 100 μM suppressed the tyrosinase activity by 15.8%, 19.2%, and 23.7%, respectively. At 300 μM, tyrosinase activity was inhibited by 25.6% (arbutin), 29.7% (β-piceid), and 64% (α-piceid). The α-piceid isomers showed better tyrosinase inhibitory activity than arbutin and β-piceid (Fig. 3).

**Fig. 4:**
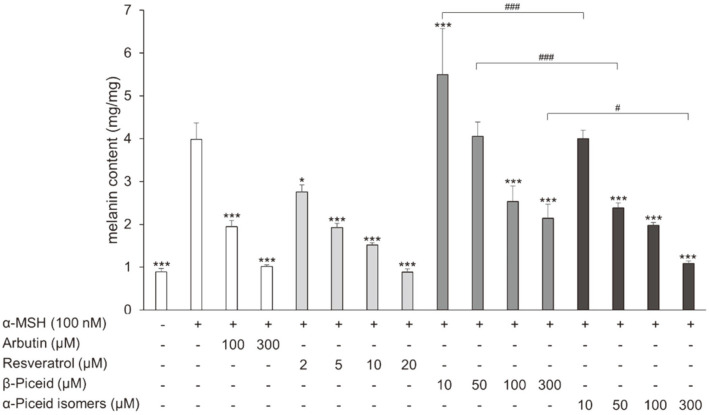
Effect of drugs on cellular melanin content in α-MSH-stimulated B16F10 melanoma cells. The cells were treated with α-MSH alone or together with arbutin, resveratrol, β-piceid, or α-piceid for 72 h and then cellular melanin content was measured. Significant differences were compared with α-MSH treated cells: *p < 0.05 and ***p < 0.001, significant differences were also observed between β-piceid and α-piceid isomers: #*p* < 0.05 and ###*p* < 0.001.

Similar to the results of tyrosinase activity, melanin synthesis was best suppressed by resveratrol in the compounds used (Fig. 4). Arbutin, β-piceid, and the α-piceid isomers suppressed melanin synthesis by 51.2%, 36.5%, and 50.4% at a concentration of 100 μM, respectively. The values were 74.5% (arbutin), 46.3% (β-piceid), and 72.7% (α-piceid isomers) at a concentration of 300 μM. The α-piceid isomers suppressed melanin synthesis more effectively than β-piceid (Fig. 4).

**Fig. 5:**
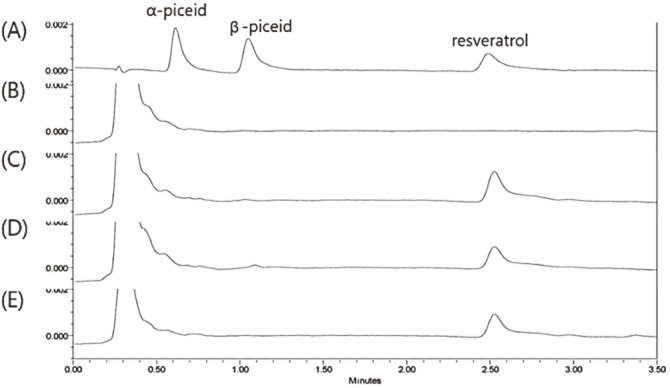
UPLC chromatograms of the cell lysates after uptake of the resveratrol and resveratrol glucosides. (A) Standard peak of 2 μM compound (mixture of α-piceid isomers, β-piceid and resveratrol), (B) a control cell lysate, (C) cell lysate 24 h after treatment of α-piceid isomers, (D) cell lysate 24 h after treatment of β-piceid, (E) cell lysate 24 h after treatment of resveratrol.

The cellular uptake of each compound was investigated in cell lysates by UPLC. Resveratrol or resveratrol glucosides were added to B16F10 cells, and the concentration of each compound present in the DMEM taken up by the B16F10 cells was then determined after 24 h (Fig. 5). The amount of piceid isomers remaining in the extracellular medium during incubation was similar with the initial amount of each compound after 24 h, whereas the amount of remaining resveratrol was only 59% of the initial amount (Fig. S5). During 24 h exposure of B16F10 cells to piceid isomers, an increase in the intracellular resveratrol content rather than that of piceid isomers was observed indicating that the piceid isomers were converted to resveratrol within the cell. In the case of resveratrol treatment, intact resveratrol was observed in the culture medium and cell lysates [17, 19].

## Discussion

Resveratrol has various beneficial effects, but its medicinal use is limited because of its low bioavailability [3-7, 10]. To synthesize highly soluble resveratrol derivatives as drugs, we reacted DGAS with resveratrol in the presence of sucrose as a donor molecule. When DGAS was used to transglycosylate salicin, baicalein, and catechin, the major transfer products were salicin glycoside, baicalein glycoside, and catechin glycoside, respectively [16, 19, 21]. Likewise, in our study, resveratrol glucosides, which are α-piceid isomers, were detected by TLC and UPLC analyses, and the chemical structures were identified by NMR analysis. The ratio of the peak areas for R1 and R2 was 5:1. Previously, a glucosyltransferase from *Phytolacca americana* (PaGT3) expressed in *E. coli* synthesized resveratrol glucosides with a ratio of *trans*-resveratrol-4′-*O*-β-glucoside to *trans*-resveratrol-3-*O*-β-glucoside of 10:3 [35]. From these data, the C-4′ position of resveratrol is the preferable site to conjugate a glucosyl residue by the transglycosylation of glycosyltransferase.

In general, glycosylated compounds have higher water solubility and chemical stability than non-glycosylated compounds [16, 19-21]. The α-piceid isomers showed 3.6 and 13.5 times higher water solubility than β-piceid and resveratrol, respectively. Interestingly, although R2 and β-piceid showed the same retention time, R2 showed higher water solubility than β-piceid. In the UPLC analysis using the C_18_ column, R1 had a shorter retention time than R2 and β-piceid, suggesting that the former is more polar than the latter. However, in the present study, R1 and R2 showed similar water solubilities, suggesting that the difference between α- and β-glucosidic bonds has a greater effect on water solubility than the position of the glucosidic linkage. The stability of polyphenols in buffered aqueous solutions at different pH and in biological fluids, such as plasma, is important for gut absorption given the sharp increase in pH from the acidic stomach to the slightly alkaline intestine. In our study, glycosylation significantly extended the half-life of resveratrol in various buffered solutions, including PBS, SBF, and DMEM. The increased stability observed in our study is consistent with the observed increased stability of baicalein with the addition of one unit of glucose [19]. Therefore, glycosylation helps to protect resveratrol from chemical and enzymatic oxidation.

Stilbenes, including resveratrol, exhibit excellent antioxidant activity, and the position of the hydroxyl residue of stilbene affects its antioxidant activity [3, 36-38]. The IC_50_ values of *trans*-resveratrol, β-piceid, and resveratrol-4′-*O*-β-glucoside were 72, 198, and 1,000 μM, respectively, indicating that the C-4′ position of stilbene plays an important role in antioxidant activity [37]. The IC_50_ value of the *cis*-form was also similar to that of the *trans*-form. Compared with the result of Waffo Teguo *et al*., the α-piceid isomers were similar to resveratrol, although the initial activities of the α-piceid isomers were low in DPPH and FRAP radical-scavenging activities. These results were due to the differences in the reaction time of antioxidant activity, the location of glycosidic linkage, or conformational differences due to α- or β-glucosidic bonds. Henríquez *et al*. reported that the extract of blackberries formed the Fe^2+^-TPTZ complex more slowly than the apple pulp [39]. In this study, β-piceid formed the Fe^2+^-TPTZ complex within 30 min, whereas the α-piceid isomers formed the complex very slowly. Whether these results are due to the type or position of the glycosidic linkage is unclear.

α-Piceid and β-piceid, which are glycoside derivatives of resveratrol, showed lower tyrosinase and melanin synthesis inhibitory capability than resveratrol. The hydroxyl group at the C-3, C-4′, and C-5 positions of resveratrol plays a critical role in tyrosinase and melanogenesis inhibitory activity [40, 41]. Therefore, the lower melanogenesis inhibitory activity of α-piceid isomer and β-piceid compared with resveratrol possibly occurred because resveratrol loses a hydroxyl group at the C-3 or C-4′ position and gains a glycosyl group. Meanwhile, the α-piceid isomers showed slightly higher activities in inhibiting tyrosinase and melanin synthesis than β-piceid. The stereochemistry of α- and β-linkage also affected the melanogenesis inhibitory activity. Funayama *et al*. examined the effect of α- and β-arbutin on the activity of tyrosinase from mushroom and mouse melanoma [42]. β-Arbutin inhibited both tyrosinase activity in mushroom and B16 mouse melanoma, whereas α-arbutin only inhibited the tyrosinase activity in the B16 mouse melanoma. The inhibitory activity of α-arbutin was 10 times as strong as that of β-arbutin. The inhibitory mechanism of α-arbutin was speculated to be a mixed-type inhibition, whereas that of β-arbutin was non-competitive. The α-piceid isomers inhibited tyrosinase activity and melanin synthesis less than resveratrol, but the isomers inhibited these processes better than β-piceid in α-MSH-stimulated B16F10 cells. The configuration of the anomeric carbon in a sugar of polyphenol glycoside may also affect the tyrosinase activity. To understand the higher tyrosinase and melanin synthesis inhibitory activity of the α-piceid isomers compared to β-piceid, we should separate each compound from the α-piceid isomer mixtures and then investigate which one is more involved in these inhibitions.

The cellular uptake analysis of resveratrol and resveratrol glucosides implied that the glycosylated form of piceid isomers was converted to resveratrol within the cells. The deglycosylation of glycosides in cells is a common phenomenon in the cellular metabolism of various glycoside compounds [17, 19]. The previous pharmacokinetic parameter results revealed that glycosylated derivatives were absorbed more rapidly and efficiently than their aglycones [43, 44].

In conclusion, resveratrol was converted to resveratrol-4′-*O*-α-glucoside and resveratrol-3-*O*-α-glucoside by DGAS. We propose that glycosylated resveratrol increased the bioavailability of resveratrol by protecting this vital molecule from chemical oxidation, thereby extending its half-life in the cell and maintaining antioxidant and melanin synthesis inhibitory properties. We anticipate that resveratrol glucosides may serve as a model for developing analogues for phytochemicals with therapeutic potential in the cosmetic industry.

## Supplemental Materials

Supplementary data for this paper are available on-line only at http://jmb.or.kr.

## Figures and Tables

**Table 1:** Stability of resveratrol and piceid in buffer solutions and cell culture medium.

		Half-life, *t*_1/2_ (h)		

		Resveratrol	β-Piceid	α-Piceid isomers
Buffer	pH 2.0	>72	>72	>72
	PBS (pH 7.4)	28	>72	>72
	SBF (pH 7.4)	44	>72	>72
	pH 9.0	1.5	>72	>72
DMEM (without FBS)		>72	>72	>72

## References

[1] Constant J (1997). Alcohol, ischemic heart disease, and the French paradox. Clin. Cardiol..

[2] Fan E, Zhang L, Jiang S, Bai Y (2008). Beneficial effects of resveratrol on atherosclerosis. J. Med. Food..

[3] Su D, Cheng Y, Liu M, Liu D, Cui H, Zhang B (2013). Comparision of piceid and resveratrol in antioxidation and antiproliferation activities *in vitro*. PLoS One.

[4] Jang M, Cai L, Udeani GO, Slowing KV, Thomas CF, Beecher CWW (1997). Cancer chemopreventive activity of resveratrol, a natural product derived from grapes. Science.

[5] Mizutani K, Ikeda K, Kawai Y, Yamori Y (1998). Resveratrol stimulates the proliferation and differentiation of osteoblastic MC3T3-E1 cells. Biochem. Biophys. Res. Commun..

[6] Newton RA, Cook AL, Roberts DW, Leonard JH, Sturm RA (2007). Post-transcriptional regulation of melanin biosynthetic enzymes by cAMP and resveratrol in human melanocytes. J. Invest. Dermatol..

[7] Fremont L (2000). Biological effects of resveratrol. Life Sci..

[8] Baxter RA (2008). Anti-aging properties of resveratrol: review and report of a potent new antioxidant skin care formulation. J. Cosmet. Dermatol..

[9] Chimento A, De Amicis F, Sirianni R, Sinicropi MS, Puoci F, Casaburi I (2019). Progress to improve oral bioavailability and beneficial effects of resveratrol. Int. J. Mol. Sci..

[10] Walle T (2011). Bioavailability of resveratrol. Ann. NY Acad. Sci..

[11] Walle T, Hsieh F, DeLegge MH, Oatis JE, Walle UK (2004). High absorption but very low bioavailability of oral resveratrol in humans. Drug Metab. Dispos..

[12] Yu C, Shin YG, Chow A, Li Y, Kosmeder JW, Lee YS (2002). Human, rat, and mouse metabolism of resveratrol. Pharm. Res..

[13] Wenzel E, Somoza V (2005). Metabolism and bioavailability of *trans*-resveratrol. Mol. Nutr. Food Res..

[14] Orsini F, Verotta L, Klimo K, Gerhäuser C (2016). Synthesis of resveratrol derivatives and in vitro screening for potential cancer chemopreventive activities. Arch. Pharm. Chem. Life Sci..

[15] Liu Q, Kim C, Jo YH, Kim SB, Hwang BY, Lee MK (2015). Synthesis and biological evaluation of resveratrol derivatives as melanogenesis inhibitors. Molecules.

[16] Cho HK, Kim HH, Seo DH, Jung JH, Park JH, Baek NI (2011). Biosynthesis of (+)^−^catechin glycosides using recombinant amylosucrase from *Deinococcus geothermalis* DSM 11300. Enzyme Microb. Technol..

[17] Moon KO, Park H, Joo M, Ha KT, Baek NI, Park CS (2017). Glycosylation enhances the physicochemical properties of caffeic acid phenethyl ester. J. Microbiol. Biotechnol..

[18] Park S, Moon K, Park CS, Jung DH, Cha J (2018). Synthesis of aesculetin and aesculin glycosides using engineered *Escherichia coli* expressing *Neisseria polysaccharea* amylosucrase. J. Microbiol. Biotechnol..

[19] Kim KH, Park YD, Park H, Moon KO, Ha KT, Baek NI (2014). Synthesis and biological evaluation of a novel baicalein glycoside as an anti-inflammatory agent. Eur. J. Pharmacol..

[20] Kometani T, Nishimura T, Nakae T, Takii H, Okada S (1996). Synthesis of neohesperidin glycosides and naringin glycosides by cyclodextrin glucano-transferase from an Alkalophilic *Bacillus* species. Biosci. Biotechnol. Biochem..

[21] Jung JH, Seo DH, Ha SJ, Song MC, Cha J, Yoo SH (2009). Enzymatic synthesis of salicin glycosides through transglycosylation catalyzed by amylosucrases from *Deinococcus geothermalis* and *Neisseria polysaccharea*. Carbohydr. Res..

[22] Park H, Kim J, Choi KH, Hwang S, Yang SJ, Baek NI (2012). Enzymatic synthesis of piceid glucosides using maltosyltransferase from *Caldicellulosiruptor bescii* DSM 6725. J. Agric. Food Chem..

[23] Regev-Shoshani G, Shoseyov O, Bilkis I, Kerem Z (2003). Glycosylation of resveratrol protects it from enzymic oxidation. Biochem. J..

[24] Moon K, Cha J (2020). Enhancement of antioxidant and antibacterial activities of *Salvia miltiorrhiza* roots fermented with *Aspergillus oryzae*. Foods.

[25] Park HJ, Lee EH, Jung HY, Kang IK, Cho YJ (2020). Effects of phenolics from *Oplismenus undulatifolius* in α-MSH-stimulated B16F10 melanoma cells. J. Appl. Biol. Chem..

[26] Marier JF, Vachon P, Gritsas A, Zhang J, Moreau JP, Ducharme MP (2002). Metabolism and disposition of resveratrol in rats: extent of absorption, glucuronidation, and enterohepatic recirculation evidenced by a linked-rat model. J. Pharmacol. Exp. Ther..

[27] Remsberg CM, Yáñez JA, Ohgami Y, Vega‐Villa KR, Rimando AM, Davies NM (2008). Pharmacometrics of pterostilbene: preclinical pharmacokinetics and metabolism, anticancer, antiinflammatory, antioxidant and analgesic activity. Phytother. Res..

[28] Thaipong K, Boonprakob U, Crosby K, Cisneros-Zevallos L, Byrne DH (2006). Comparison of ABTS, DPPH, FRAP, and ORAC assays for estimating antioxidant activity from guava fruit extracts. J. Food Compos. Anal..

[29] Jun SY, Park KM, Choi KW, Jang MK, Kang HY, Lee SH (2008). Inhibitory effects of arbutin-β-glycosides synthesized from enzymatic transglycosylation for melanogenesis. Biotechnol. Lett..

[30] Lee TH, Seo JO, Baek SH, Kim SY (2014). Inhibitory effects of resveratrol on melanin synthesis in ultraviolet B-induced pigmentation in Guinea pig skin. Biomol. Ther..

[31] Yoon HS, Hyun CG, Lee NH, Park SS, Shin DB (2016). Comparative depigmentation effects of resveratrol and its two methyl analogues in α-melanocyte stimulating hormone-triggered B16/F10 murine melanoma cells. Prev. Nutr. Food Sci..

[32] Kim JK, Park KT, Lee HS, Kim M, Lim YH (2012). Evaluation of the inhibition of mushroom tyrosinase and cellular tyrosinase activities of oxyresveratrol: comparison with mulberroside A. J. Enzyme Inhib. Med. Chem..

[33] Satooka H, Kubo I (2012). Resveratrol as a *k*_cat_ type inhibitor for tyrosinase: potentiated melanogenesis inhibitor. Bioorg. Med. Chem..

[34] Jeong ET, Jin MH, Kim MS, Chang YH, Park SG (2010). Inhibition of melanogenesis by piceid isolated from *Polygonum cuspidatum*. Arch. Pharm. Res..

[35] Ozaki S, Imai H, Iwakiri T, Sato T, Shimoda K, Nakayama T (2012). Regioselective glucosidation of trans-resveratrol in *Escherichia coli* expressing glucosyltransferase from *Phytolacca americana*. Biotechnol. Lett..

[36] Stivala LA, Savio M, Carafoli F, Perucca P, Bianchi L, Maga G (2001). Specific structural determinants are responsible for the antioxidant activity and the cell cycle effects of resveratrol. J. Biol. Chem..

[37] Waffo Teguo P, Fauconneau B, Deffieux G, Huguet F, Vercauteren J, Mérillon J-M (1998). Isolation, identification, and antioxidant activity of three stilbene glucosides newly extracted from Vitis vinifera cell cultures. J. Nat. Prod..

[38] Fang JG, Lu M, Chen ZH, Zhu HH, Li Y, Yang L (2002). Antioxidant effects of resveratrol and its analogues against the free‐radical‐induced peroxidation of linoleic acid in micelles. Chem. Eur. J..

[39] Henríquez C, López-Alarcón C, Lutz MGM, Speisky H (2011). Time-dependence of ferric reducing antioxidant power (FRAP) index in Chilean apples and berries. Arch. Latinoam. Nutr..

[40] Ohguchi K, Tanaka T, Kido T, Baba K, Iinuma M, Matsumoto K (2003). Effects of hydroxystilbene derivatives on tyrosinase activity. Biochem. Biophys. Res. Commun..

[41] Lee HS, Lee BW, Kim MR, Jun JG (2010). Syntheses of resveratrol and its hydroxylated derivatives as radical scavenger and tyrosinase inhibitor. Bull. Korean Chem. Soc..

[42] Funayama M, Arakawa H, Yamamoto R, Nishino T, Shin T, Murao S (1995). Effects of α-and β-arbutin on activity of tyrosinases from mushroom and mouse melanoma. Biosci. Biotechnol. Biochem..

[43] Yamada M, Tanabe F, Arai N, Mitsuzumi H, Miwa Y, Kubota M (2006). Bioavailability of glucosyl hesperidin in rats. Biosci. Biotechnol. Biochem..

[44] Jiang JR, Yuan S, Ding JF, Zhu SC, Xu HD, Chen T (2008). Conversion of puerarin into its 7-*O*-glycoside derivatives by *Microbacterium oxydans* (CGMCC 1788) to improve its water solubility and pharmacokinetic properties. Appl. Microbiol. Biotechnol..

