# Supplementary material for: Enzymatic Synthesis of Resveratrol α-Glucoside by Amylosucrase of Deinococcus geothermalis
Source: J Microbiol Biotechnol. 2021 Sep 26;31(12):1692–700. doi: 10.4014/jmb.2108.08034 (PMC9706033; doi:10.4014/jmb.2108.08034)
Supplement: Supplementary file 1 [file jmb-31-12-1692-supple.pdf]

Supplementary data

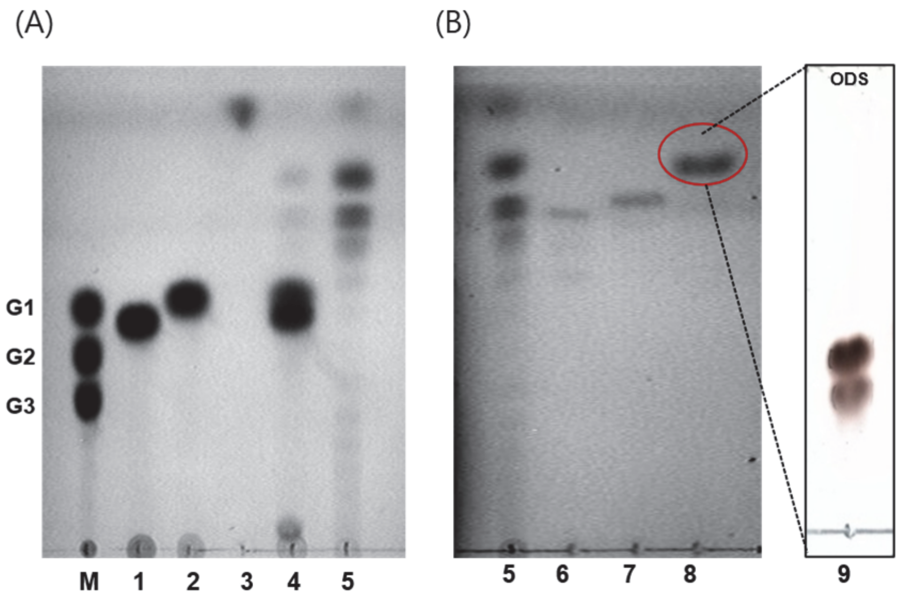

Fig. S1. TLC analysis of resveratrol and resveratrol glucosides. (A) TLC analysis using silica gel 60 of the reaction products of DGAS with resveratrol as a receptor and sucrose as a donor. Lane M, maltooligosaccharide standard containing glucose (G1) through maltotriose (G3); lane 1, sucrose; lane 2, fructose; lane 3, resveratrol; lane 4, resveratrol and sucrose reaction products; lane 5: reaction products after sugar removal by C_{18}-T cartridge, (B) TLC analysis of the resveratrol transfer products by the transglycosylation of DGAS after purification. Lane 5: after sugar removal by C_{18}-T cartridge, lane 6, 7, 8, the three transfer products; lane 9: after separation of the major products by ODS TLC plate (silica gel RP-18 F254S).

(A)

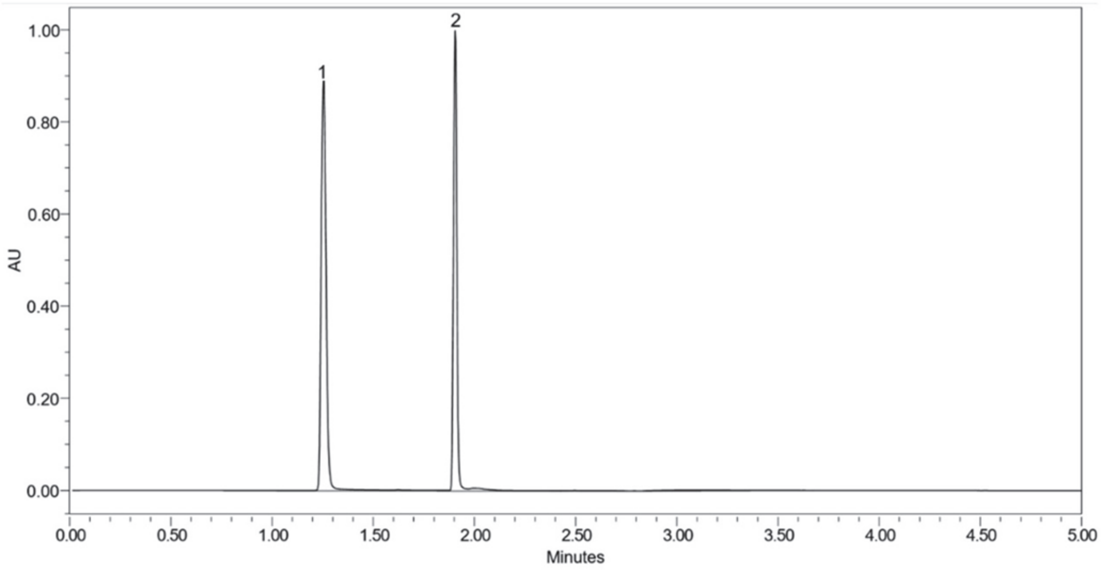

(B)

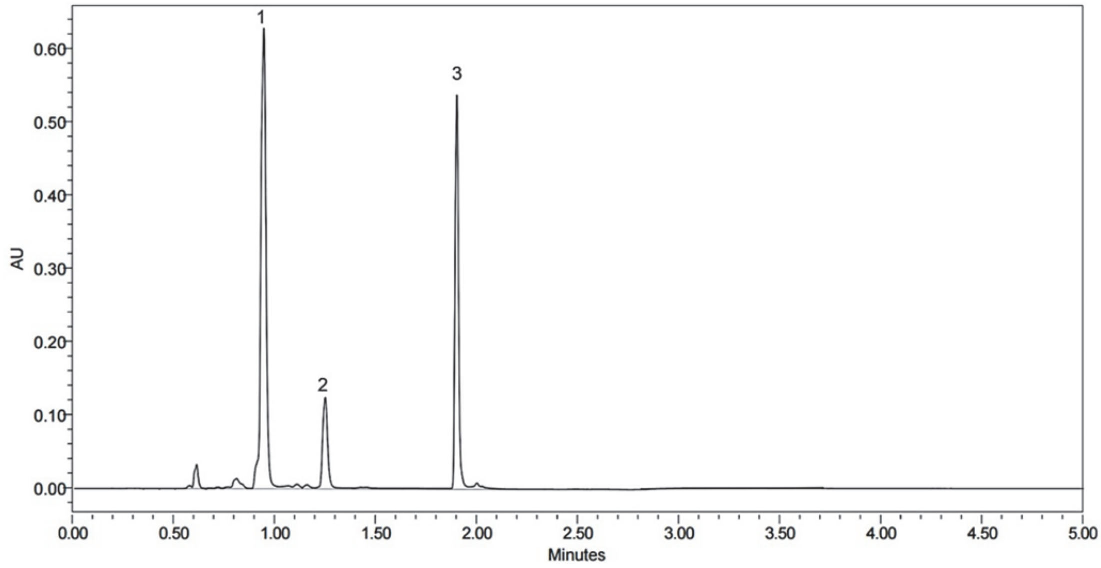

Fig. S2. UPLC analysis of the resveratrol and resveratrol glucosides. (A) Standard peak of β-piceid and resveratrol. Peak 1, resveratrol-3-*O*-β-glucoside; peak 2, resveratrol. (B) peak 1, resveratrol-4'-*O*-α-glucoside; peak 2, resveratrol-3-*O*-α-glucoside; peak 3, resveratrol.

(A) DPPH radical scavenging activity

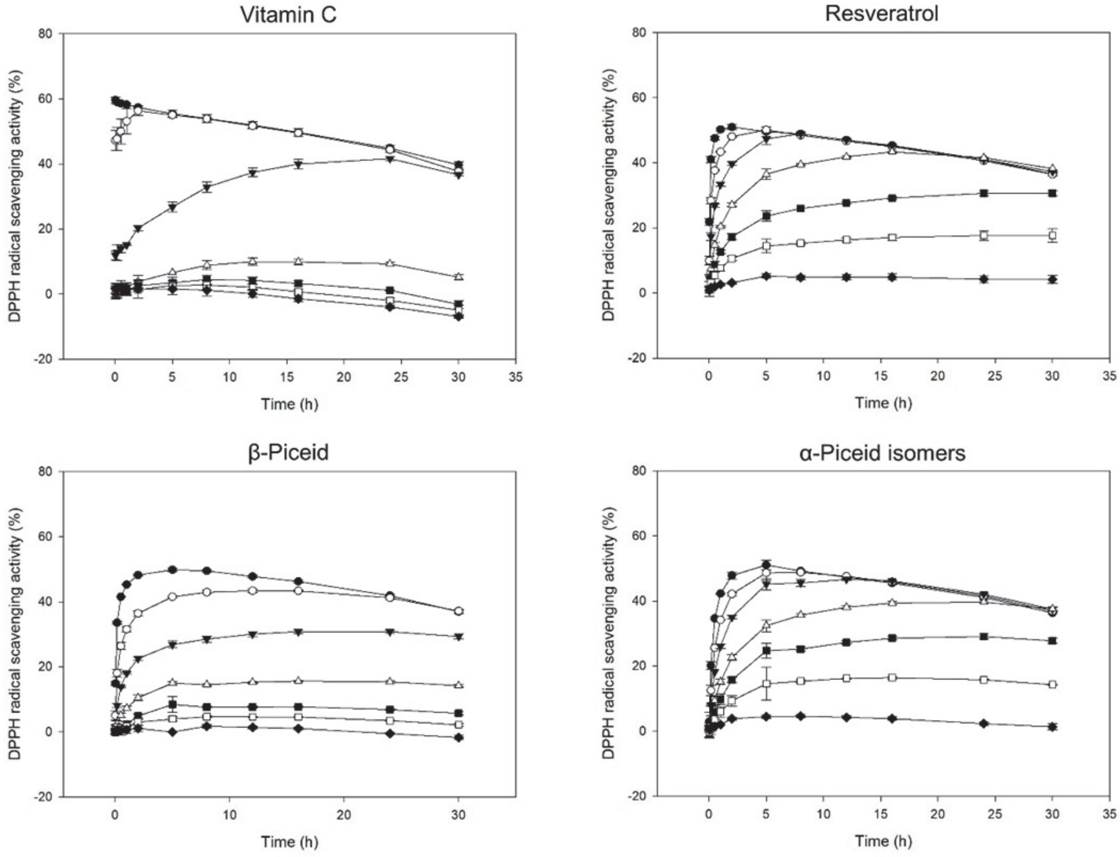

(B) FRAP radical scavenging activity

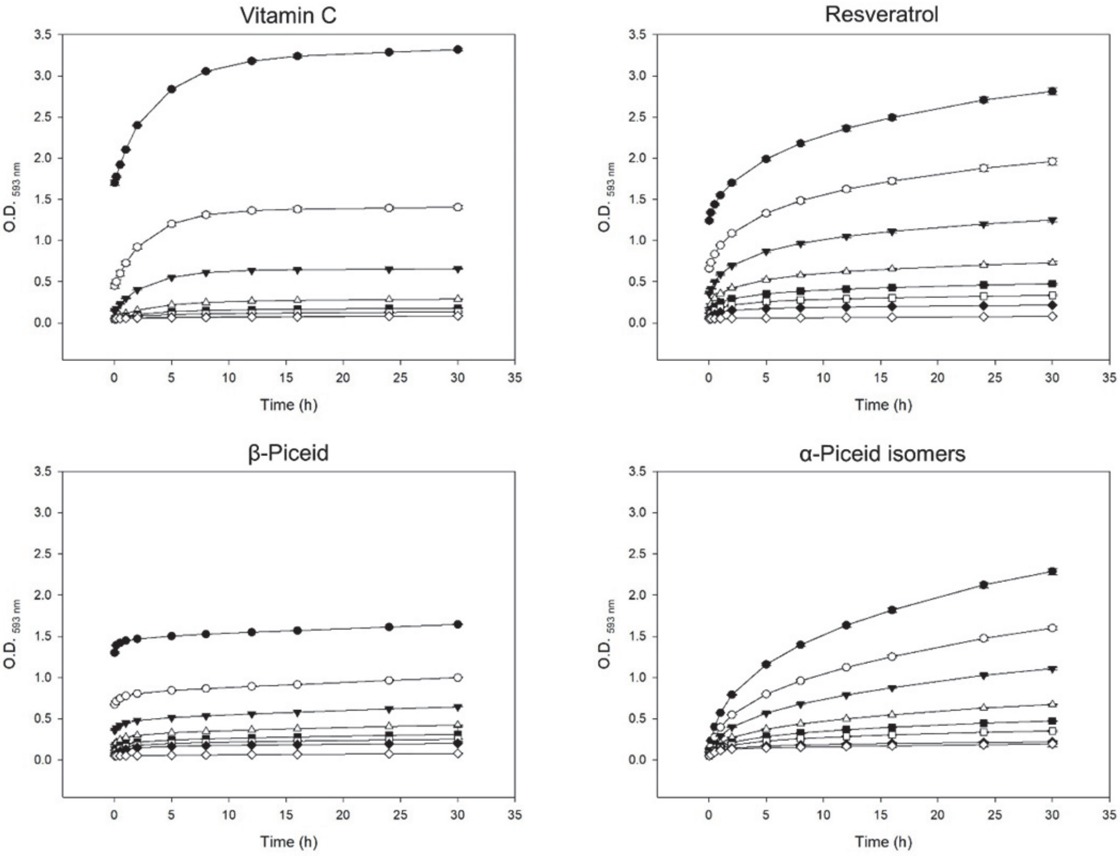

(C) Hydroxyl radical scavenging assay

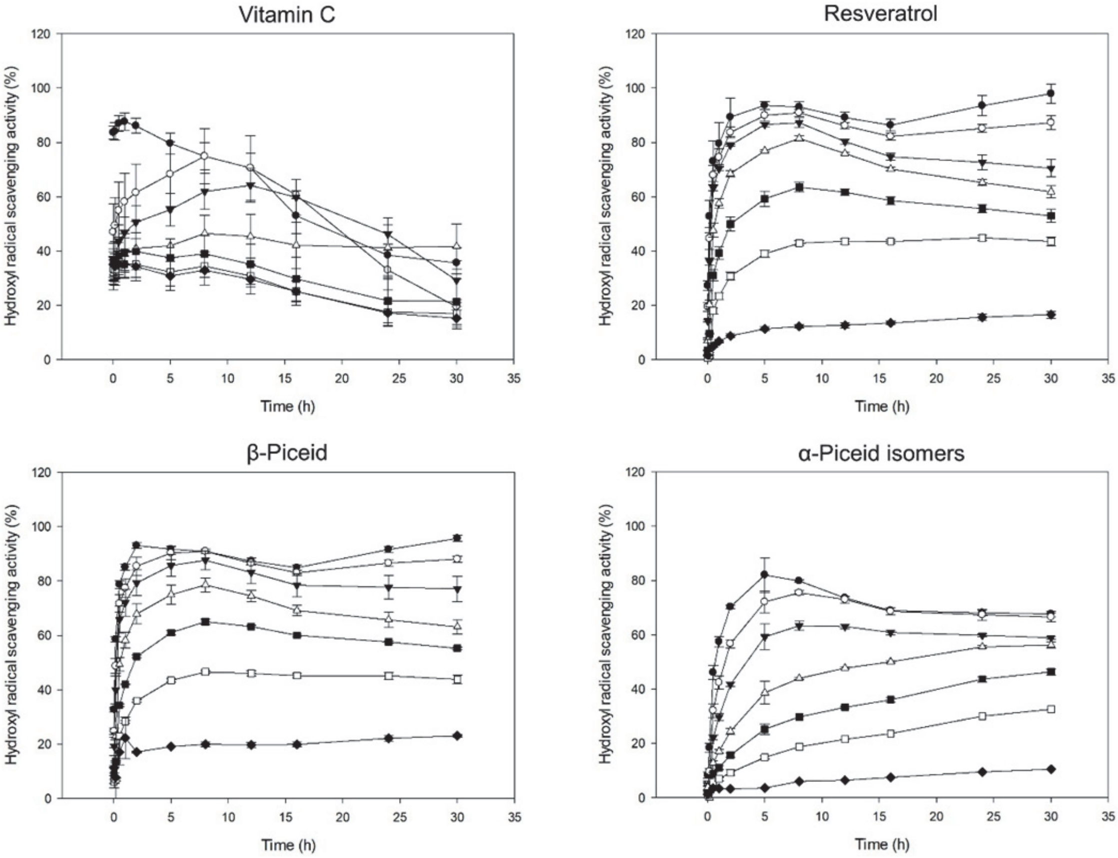

Fig. S3. Determination of antioxidant activity of vitamin C, resveratrol, β-piceid, and α-piceid isomers by the (A) DPPH, (B) FRAP, and (C) hydroxyl radical scavenging method using different concentrations (2 mM (filled circle), 1 mM (open circle), 0.5 mM (filled triangle), 0.2 mM (open triangle), 0.1 mM (filled square), 0.05 mM (open square), 0.01 mM (filled diamond), and 0 mM (open diamond)). Values are expressed as mean ± standard deviation from triplicates.

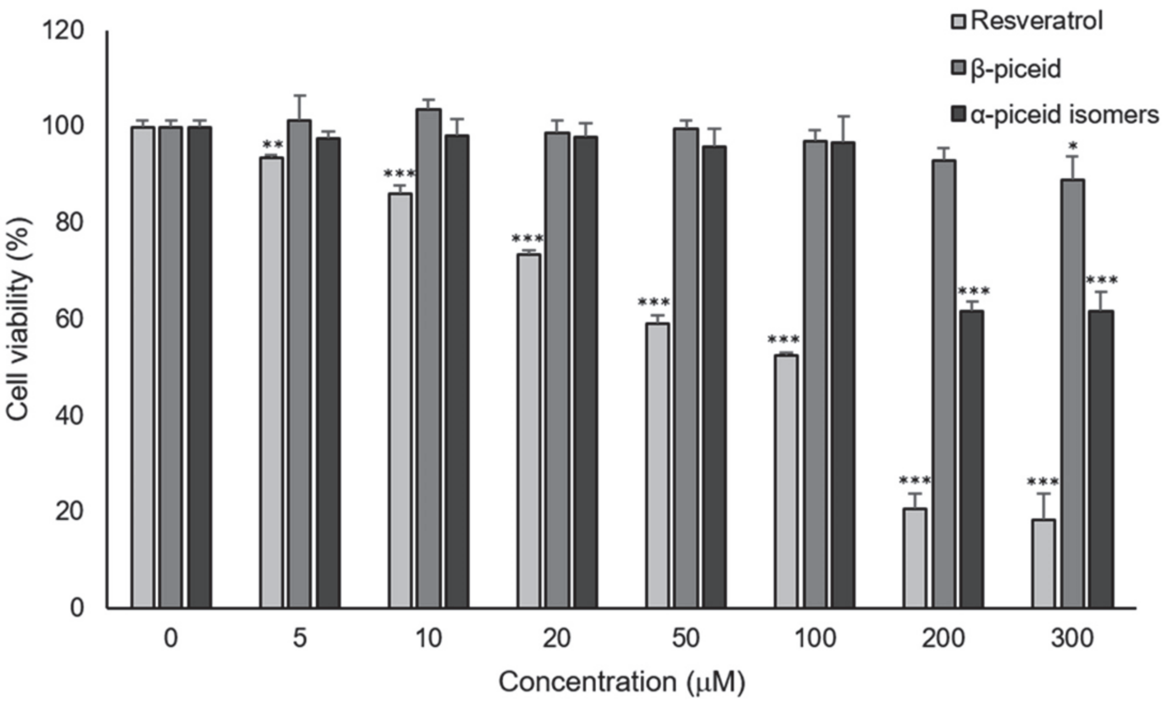

Fig. S4. Effect of resveratrol, β-piceid and α-piceid isomers on the viability of B16F10 cells. The cells were treated with the compounds for 24 h and then cell viability was measured by MTT assay. Significant differences compared with non-treated cells. ** $p < 0.005$ and *** $p < 0.001$.

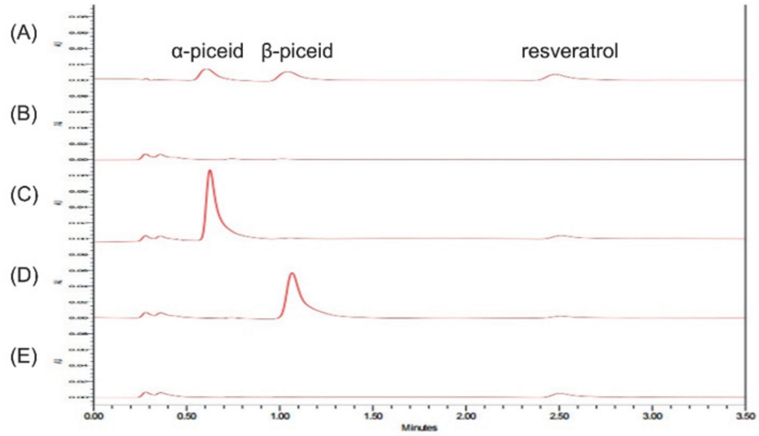

Fig. S5. UPLC chromatogram of the cell media after treatment of the resveratrol and resveratrol glucosides. (A) standard peak of 20 μM compound (mixture of α-piceid isomers, β-piceid and resveratrol), (B) a control cell media, (C) cell media after 24 h treatment of α-piceid isomers, (D) cell media after 24 h treatment of β-piceid, (E) cell media after 24 h treatment of resveratrol.
